# Age, Diet and Epidermal Signaling Modulate Dermal Fibroblasts’ Adipogenic Potential

**DOI:** 10.3390/ijms21238955

**Published:** 2020-11-25

**Authors:** Katarzyna Walendzik, Joanna Bukowska, Marta Kopcewicz, Sylwia Machcinska, Jeffrey M. Gimble, Barbara Gawronska-Kozak

**Affiliations:** 1 Institute of Animal Reproduction and Food Research, Polish Academy of Sciences, 10-748 Olsztyn, Poland; k.walendzik@pan.olsztyn.pl (K.W.); j.bukowska@pan.olsztyn.pl (J.B.); m.kopcewicz@pan.olsztyn.pl (M.K.); s.machcinska@pan.olsztyn.pl (S.M.); 2 LaCell LLC, New Orleans, LA 70112, USA; jeffrey.gimble@obatalasciences.com; 3 Obatala Sciences Inc., 2000 Lakeshore Drive, #4020, New Orleans, LA 70148, USA; 4 Departments of Medicine, Structural and Cellular Biology, and Surgery and Center for Stem Cell Research and Regenerative Medicine, Tulane University School of Medicine, New Orleans, LA 70118, USA

**Keywords:** dermal white adipose tissues, age, diet, dermal fibroblasts, Foxn1

## Abstract

The recognition of a distinct fat depot, the dermal white adipose tissue (dWAT), points out the complexity of the interaction among skin resident cells: keratinocytes, dermal fibroblasts (DFs) and adipocytes in response to physiological (diet, age) and pathological (injury) stimulations. dWAT has been recognized as a significant contributor to thermoregulation, hair cycle, immune response, wound healing and scarring. In this study, we examined age- and diet-related changes in dWAT modulation and DFs’ adipogenic potential. The data showed that diet modulates dWAT expansion predominantly by hypertrophy, whereas age affects the pool of adipocyte progenitor cells in the skin indicating its role in dWAT hyperplasia. Analysis of DFs’ migratory abilities in the model of skin explants isolated from the skin of young, old, low (LFD)- or high (HFD)-fat diet C56BL/6 mice revealed that HFD, regardless of animal age has the most profound stimulatory impact of DF migration. We determined that the adipogenic potential of DFs is comparable to stromal vascular fraction (SVF) of inguinal fat depot and ear mesenchymal stem cells (EMSC). We also showed the stimulatory role of epidermally expressed transcription factor Foxn1 on adipogenic signaling: bone morphogenetic protein 2 (Bmp2) and insulin-like growth factor 2 (Igf2) in keratinocytes.

## 1. Introduction

Mammalian skin is composed of three layers: epidermis, dermis and hypodermis. Epidermis is built up by stratified keratinocytes separated from the underlying dermis by a basement membrane. Dermis is comprised of both a fibroblast-rich dermis and an underlying dermal white adipose tissue (dWAT). Hypodermis, formed by connective tissues and adipose tissues, in rodents is separated from the dermis by a layer of striated muscle (*panniculus carnosus*). dWAT forms the adipose compartment within the dermis which is primarily composed of adipocytes defined as intradermal adipocytes [1]. This unique population of fat cells actively participates in the various physiological and pathological processes in the skin. A decrease in the ambient temperature increases the thickness of the dWAT layer, which indicates the possible role of intradermal adipocytes in generating heat and maintaining a constant temperature, thus participating in thermoregulation [2]. Studies by Festa et al. showed that the resident intradermal adipocytes contribute to follicular stem cell activation and control hair follicle regeneration by producing platelet-derived growth factor (PDGF) signals [3]. dWAT was additionally identified as contributor in the immune defense response induced by bacterial infections of the skin [4]. It was also delineated that resident intradermal adipocytes participate in the skin wound healing process by promoting fibroblast production and increasing their migration into the wound bed [5]. Moreover, loss of dWAT is observed in murine models of dermal fibrosis, whereas a lack of dWAT is characteristic for patients with systematic sclerosis (SSc; scleroderma) [6]. Treatment with bleomycin (an inducer of dermal fibrosis in mice), resulted in the reduction of the dWAT layer and a decrease in the levels of adipogenic markers (Pparγ2, Fabp4, AdipoQ) in C57BL/6J mice [7]. Further research using aP2-Cre–transgenic mice treated with bleomycin showed that loss of intradermal adipocytes leads to dermal fibrosis [7]. However, despite increasing research interest in the role of dWAT in skin physiology, the current knowledge regarding dWAT homeostasis and the molecular pathways which modulate its physiology is limited. One possible factor involved in dWAT regulation is the epidermal Wnt/β-catenin signaling pathway. Donati et al. demonstrated that 3T3-L1 preadipocytes stimulated by adipogenic media and cultured in keratinocytes conditioned media from K14Cre/CatnbFlox(ex3)/+ mice, in which K14-positive epidermal basal keratinocytes express β-catenin, differentiate into adipocytes more efficiently than those cultured in keratinocyte-conditioned media from wild type mice [8]. The authors concluded that adipocyte differentiation was induced by epidermal Wnt/β-catenin through secretion of ligands for the Bmp and insulin signaling pathways [8].

Another factor that may participate in dWAT homeostasis is the transcription factor Foxn1. In the skin, epidermally expressed Foxn1 is responsible for the initiation of the keratinocytes’ terminal differentiation program (suprabasal layer of epidermis) and stimulation of epithelial cell proliferation (basal layer) [9,10]. Previous studies have reported the regulatory role of Foxn1 in the reparative (scar-forming) skin wound healing by participation in re-epithelialization and by promoting epithelial to mesenchymal transition (EMT) [11,12]. Our recent data demonstrated that Foxn1 may regulate susceptibility to diet-induced obesity. Indeed, activation/nonactivation of Foxn1 may play a role in modulating body weight/metabolic homeostasis [13]. Moreover, partial inactivation of Foxn1 abrogated adipogenesis in intact and injured skin by possible impact on the expression of adipogenesis-promoting genes Bmp2 and Igf2 [13].

In the present study, we examined how age and diet (1) affect the adipogenic potential of DFs and (2) modulate the dWAT compartment in the skin. We also investigated the potential role of epidermally expressed Foxn1 on DF adipogenic potential through Bmp2 and Igf2 signaling contribution using the coculture model of keratinocytes and differentiated DFs.

## 2. Results

### 2.1. Dermal Fibroblasts from Mice Fed a High-Fat Diet Display Increased Migratory Capability

**Figure 1:**
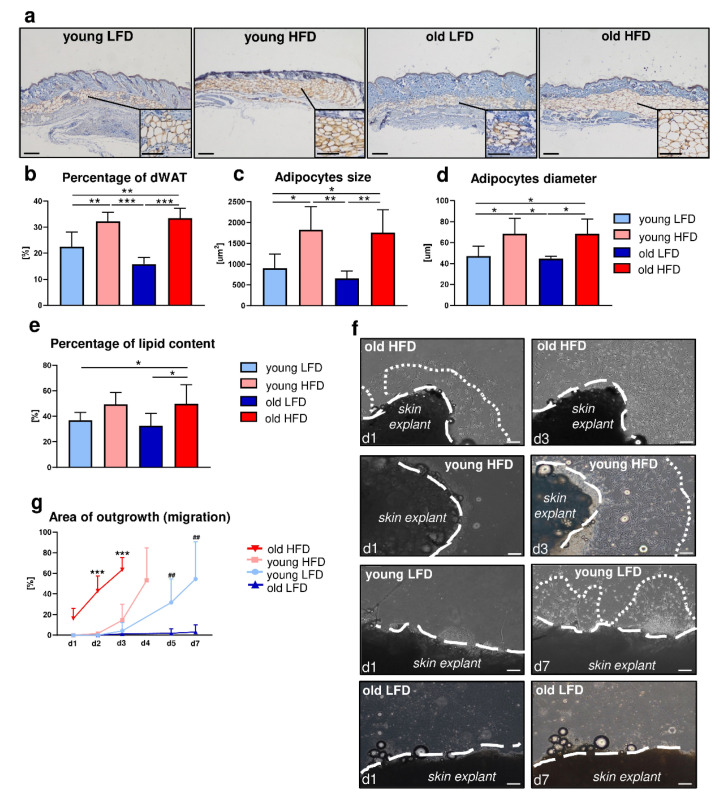
Morphological and functional: age- and diet-related changes in dermal white adipose tissue (dWAT) and migration of dermal fibroblasts (DFs). (**a**) Immunohistochemical detection of perilipin in the histological skin sections from young low-fat diet (LFD), young high-fat diet (HFD), old LFD and old HFD B6 mice. Quantitative analyses of: (**b**) percentage of dWAT in relation to the total skin area, (**c**) dWAT adipocyte size, (**d**) dWAT adipocyte diameter and (**e**) percentage of lipid content in skin. Values are the mean ± SD. Asterisks indicate significant differences (* *p* < 0.05; ** *p* < 0.01; *** *p* < 0.001). (**f**) Representative images of DF migration from skin explants isolated from young, old, LFD or HFD B6 mice (*n* = 3 mice per group, *n* = 10 explants per each mice). Images were taken at day 1 (d1), 3 (d3) or 7 (d7) of culture. Dashed lines indicate the edges of the skin explants, dotted lines depict the area of DF extended migration. (**g**) Quantification of the area of DF outgrowth (migration). Values are the mean ± SD. Asterisks indicate significant differences between the old HFD group vs. the young HFD, young LFD and old LFD at day 2 (d2) and day 3 (d3) of culture (*** *p* < 0.001). Hashes indicate significant differences between young LFD vs. old LFD at day 5 (d5) and day 7 (d7) (## *p* < 0.01). Scale bar (**a**) 200 μm, insets (**a**) 100 μm, (**f**) 200 μm.

Our recent studies revealed that the obesogenic environment (HFD) modulates dWAT [14]. We observed that the decrease in fibroblast-rich dermis thickness due to age is compensated by a thickened layer of dWAT in old, HFD-fed animals [13,14]. To further address the impact of diet and/or age on dWAT, we performed histological assessment of adipocytes’ morphometry in the skin of young (4–5 months old) relative to old (20–21months old) C57BL/6 (B6) mice fed either LFD or HFD. Immunohistochemical detection of perilipin, the protein associated with the surface of lipid droplets, revealed the presence of an adipocyte-rich layer deposited between the fibroblast-rich dermis and *panniculus carnosus* that corresponds to dWAT (Figure 1a).

Measurement of the percentage of the adipocyte-rich region in relation to the total skin area (comprising epidermis and dermis) showed an increase in adiposity in animals fed HFD regardless of age (Figure 1b). The differences in dWAT expansion between mice fed LFD or HFD were additionally reflected by the analysis of the individual adipocyte size (Figure 1c) and diameter (Figure 1d). Animals fed HFD had a greater number of large adipocytes (Figure 1c) with a larger diameter (Figure 1d) relative to mice fed LFD, regardless of age. Furthermore, lipids extracted from the skin tissues revealed a higher percentage of lipid content collected from the mice fed HFD especially in the group of old animals (Figure 1e).

Prior work by Schmidt and Horsley showed that the presence of intradermal adipocytes stimulates DF proliferation and their ability to migrate towards wounded areas during the healing process [5]. To evaluate the impact of diet and/or age on the migratory abilities of DFs, we performed ex vivo experiments in which skin tissues were collected from young and old B6 mice, fed either LFD or HFD and cultured as a skin explants (Figure 1f,g, Supplementary Table S1). Daily, for a period of 7 days, microscopic evaluation of DF migration from skin explants revealed the greatest migratory ability of DFs originated from the skin of old and HFD-fed mice where full outgrowth was apparent by day 3 of culture (Figure 1f,g). Likewise, a significant increase in the migratory ability of DFs was also detected in the skin explants collected from young mice fed HFD with full outgrowth detected by day 4 (Figure 1f,g). In contrast, skin explants from young LFD mice only displayed complete outgrowth of cells by day 7. It was noteworthy that DFs isolated from old mice fed LFD cultured under the same conditions as skin explants from other groups showed no migratory abilities whatsoever during the entire course of the experiment (Figure 1f,g).

### 2.2. Age More Than Diet Determines the Adipogenic Potential of DFs

**Figure 2:**
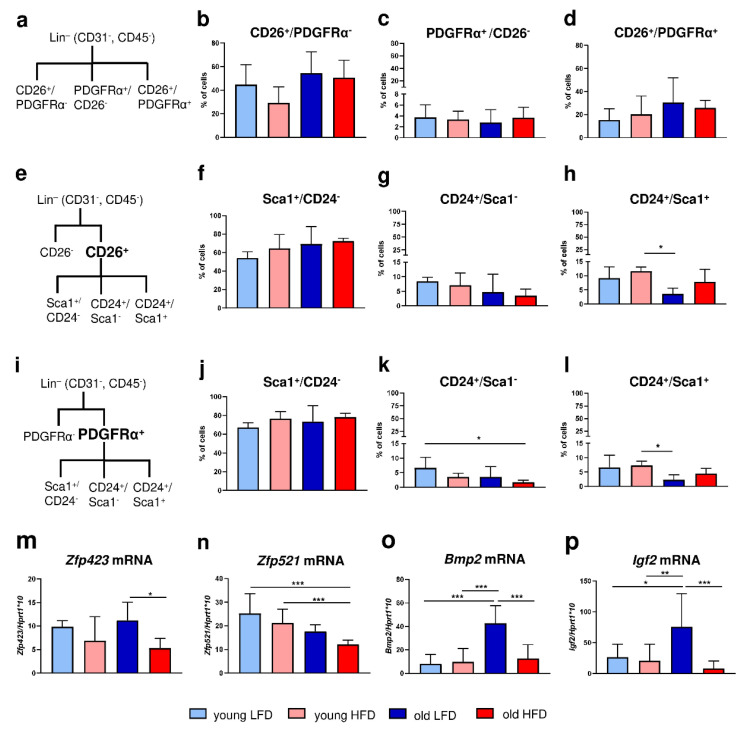
Phenotypic characteristics of DFs isolated from young, old, LFD or HFD B6 mice. (**a**) A scheme of flow cytometry analysis for CD26 or PDGFRα within the Lin^−^ (CD31^−^CD45^−^) population. The analysis of (**b**) CD26^+^ PDGFRα^−^, (**c**) CD26^−^PDGFRα^+^, (**d**) CD26^+^ PDGFRα^+^ cells. (**e**) A scheme of flow cytometry analysis for CD26 population within the Lin^−^ (CD31^−^CD45^−^) population. The analysis of (**f**) Sca1^+^ CD24^−^, (**g**) CD24^+^ Sca1^−^, (**h**) CD24^+^ Sca1^+^ cells within the CD26^+^ population. (**i**) A scheme of flow cytometry analysis for PDGFRα population within the Lin^−^ (CD31^−^CD45^−^) population. The analysis of (**j**) Sca1^+^ CD24^−^, (**k**) CD24^+^ Sca1^−^, (**l**) CD24^+^ Sca1^+^ cells within the PDGFRα^+^ population. (**m**) *Zfp423*, (**n**) *Zfp521*, (**o**) *Bmp2*, and (**p**) *Igf2* qRT-PCR analysis of mRNA expression in the isolated DFs. Values are the mean ± SD. Asterisks indicate significant differences (* *p* < 0.05; ** *p* < 0.01; *** *p* < 0.001).

Given that HFD stimulated hypertrophy of adipocytes within dWAT (see Figure 1a–d), increased lipid content in the skin (see Figure 1e) and accelerated DF migratory ability (see Figure 1f,g), we next performed phenotypic characterization of the cells isolated from the skin of young or old mice fed either LFD or HFD based on flow cytometry (Figure 2, Supplementary Figures S1–S3).

Initially, we distinguished two populations of DFs: (1) CD26^+^ (CD31^−^/CD45^−^) that were characteristic for the upper dermis and postulated to have proscarring/profibrotic potential [15,16] and (2) PDGFRα^+^ (CD31^−^/CD45^−^) that were committed to the adipocyte lineage [3,17] (Figure 2a). The majority of skin cells were CD26^+^, regardless of the animals’ age or diet (Figure 2a–d, Supplementary Figure S1). DFs isolated from the skin of each experimental group had a ten-fold-greater percentage of cells expressing the CD26 marker (young LFD: 44.68 ± 17.05; young HFD: 29.23 ± 13.56; old LFD: 54.46 ± 18.05; old HFD: 50.6 ± 14.84) compared to the expression of PDGFRα (young LFD: 3.74 ± 2.28; young HFD: 3.33 ± 1.53; old LFD: 2.78 ± 2.35; old HFD: 3.68 ± 1.92). Although statistical significance was not achieved, the percentage of CD26^+^ cells was higher in the population of fibroblasts isolated from mice fed LFD in comparison to mice fed HFD especially in the group of young animals (Figure 2b). No differences in the percentage of cells expressing PDGFRα^+^/CD26^−^ or PDGFRα^+^/CD26^+^ were observed among examined groups of mice (Figure 2c,d).

Next, we separately analyzed the population of PDGFRα^+^ or CD26^+^ cells based on co-expression of CD24 and/or Sca1; both are known as markers of adipocyte progenitor/precursor cells [3,18,19] (Figure 2e,i, Supplementary Figures S2 and S3). Despite the large differences in cell abundance between CD26^+^ and PDGFRα^+^ populations the percentage of Sca1^+^ (Figure 2f,j), CD24^+^ (Figure 2g,k) or Sca^+^/CD24^+^ (Figure 2h,l) cells within either PDGFRα^+^ (Figure 2f,g) or CD26^+^ (Figure 2j,l) cell populations was alike. More than 50% of cells were Sca1^+^, regardless of the animals’ age or diet (Figure 2f,j). The percentage of CD24^+^ cells (Figure 2g,k) decreased with animal age, particularly in old HFD mice. Likewise, the double-positive CD24^+^/Sca1^+^ cell population displayed a similar pattern: a decrease in percentage as a function of animal age and this was most apparent in old LFD mice (Figure 2h,l).

To further characterize the adipogenic potential of DFs in the context of age and/or diet we assessed the expression of two zinc finger transcription factors Zfp423 and Zfp521 recognized as regulators of adipose commitment and adipogenic differentiation, respectively and two growth factors recognized as adipogenic signal transduction pathways, Bmp2 and Igf2 [8,20,21]. The expression of *Zfp423* mRNA was downregulated in the DFs isolated from the mice fed HFD, regardless of the animals’ age, although the statistical significance was detected only for old HFD mice (Figure 2m). In contrast, the level of *Zfp521* mRNA expression decreased with age, being particularly low in DFs isolated from old animals fed HFD (Figure 2n). The mRNA levels of adipogenesis-promoting signal transduction factors *Bmp2* and *Igf2* showed a similar pattern of expression (compare Figure 2o,p). The highest expression of *Bmp2* and *Igf2* mRNA was detected in DFs isolated from old LFD mice and was relatively low and stable among all other groups (Figure 2o,p).

### 2.3. Adipogenic Potential of Dermal Fibroblasts Is Comparable to Adipogenic Differentiation Capabilities of Established Primary Cell Models: Ear Mesenchymal Stem Cells (EMSC) and Stromal Vascular Fraction (SVF) of Inguinal Fat Depot Models

**Figure 3:**
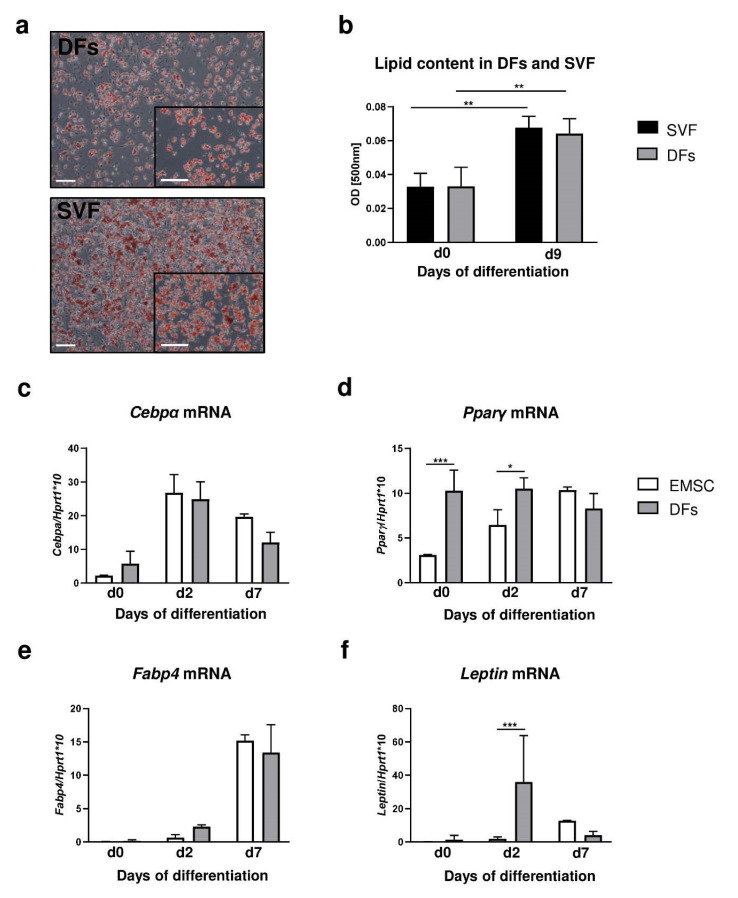
DF adipogenic differentiation potential in comparison to stromal vascular fraction (SVF) of inguinal fat depot and ear mesenchymal stem cells (EMSC). (**a**) Representative images of Oil-Red-O-stained DFs or SVF cultures at day 9 of adipogenic differentiation. (**b**) Evaluation of lipid content in differentiated DFs and SVF was performed using spectrophotometric analysis. mRNA expression of (**c**) *Cebpα*, (**d**) *Pparγ*, (**e**) *Fabp4* and (**f**) *leptin* in EMSC and DFs during adipogenic differentiation. Values are the mean ± SD. Asterisks indicate significant differences (* *p* < 0.05; ** *p* < 0.01; *** *p* < 0.001). Scale bar (**a**) 200 μm, insets (**a**) 100 μm.

To assess the adipogenic potential of DFs, it was compared to two established models of adipogenic differentiation: primary culture of preadipocytes from a stromal vascular fraction of fat depot (SVF) [22] and ear mesenchymal stem cells (EMSC) [23]. The cells from the skin (DFs), inguinal fat depot (SVF) and external ears (EMSC) of B6 mice were isolated and cultured under identical conditions (Figure 3).

Adipogenic stimulation in the presence of isobutyl-methylxanthine, dexamethasone and insulin for periods of 7 days determined that the adipogenic potential of DFs was comparable to that of both SVF (Figure 3a,b) and EMSC (Figure 3c–f). Comparison between adipogenic potential of DFs with those of SVF was based on histochemical analysis of Oil Red O staining of lipid droplets induced as a function of adipogenic stimulation (Figure 3a,b). DFs and SVF showed similar levels of lipid accumulation by day 9 at the conclusion of the differentiation period (Figure 3a). Quantification of the absorbance level of extracted Oil Red O determined that the adipogenic potential of SVF and DFs was comparable after 9 days of the differentiation process (Figure 3b).

Next, we compared the relative expression of adipogenic genes *Cebpα*, *Pparγ*, *Fabp4* and *leptin* during adipogenic stimulation (days 0 to 7) in differentiated EMSC and DFs (Figure 3c–f). For EMSC the highest levels of gene expression associated with the initial stage of differentiation. *Cebpα* (Figure 3c) and *Pparγ* (Figure 3d) were observed at day 2 of the adipogenic process whereas genes involved in the maturation of adipocytes, *Fabp4* (Figure 3e) and *leptin* (Figure 3f), showed peak expression at day 7 of adipogenesis. DFs presented a pattern similar to EMSC of the increase in *Cepbα* (Figure 3c) and *Fabp4* (Figure 3e) mRNA expression. However, DFs presented higher levels of *Pparγ* mRNA expression prior to adipogenic stimulation (day 0; *p* < 0.001) and at day 2 of differentiation (*p* < 0.05) in comparison to EMSC (Figure 3d). Additionally, *leptin* mRNA levels at day 2 of the adipogenic process were significantly higher in the DFs than in the EMSC (Figure 3f; *p* < 0.001).

### 2.4. Age, Diet and Epidermal Signaling Impact DF Adipogenic Potential

**Figure 4:**
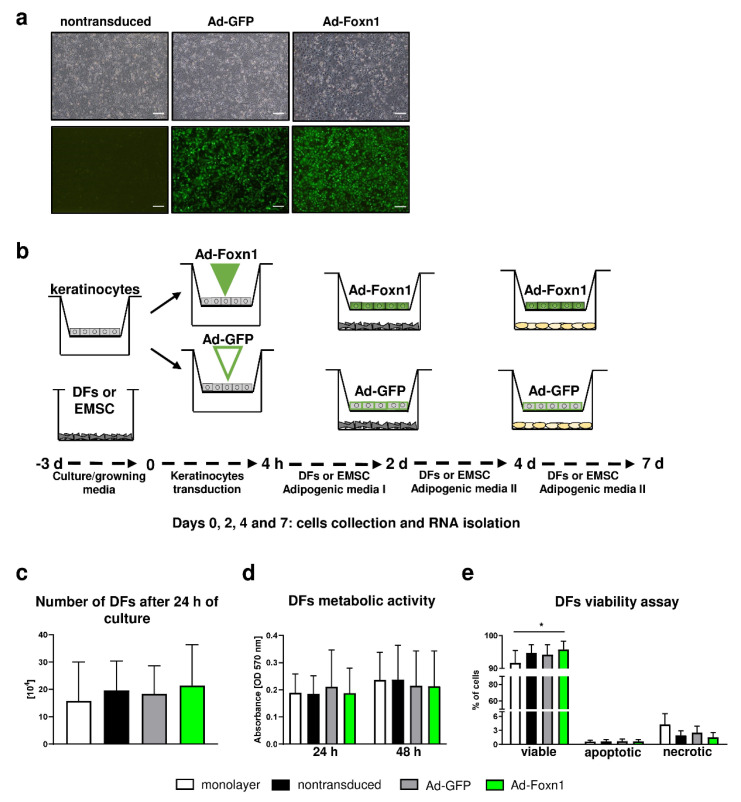
Keratinocytes-DFs coculture model for adipogenic DF differentiation. (**a**) Representative images of nontransduced keratinocytes and keratinocytes transduced with Ad-Foxn1 or Ad-GFP; bright field upper panel; fluorescent eGFP—lower panel. (**b**) Schematic presentation of the experiment course. (**c**) Trypan Blue dye exclusion, (**d**) MTT and (**e**) flow cytometry viability assays performed on DFs in different culture conditions. Values are the mean ± SD. Asterisks indicate significant differences (* *p* < 0.05). Scale bar (**a**) 200 μm.

In the skin, crosstalk between keratinocytes and DFs controls and regulates homeostasis within the skin. The interplay between keratinocytes and DFs includes direct cell-to-cell communication as well as the production of soluble factors that display autocrine and paracrine action and cell-matrix interaction [24,25]. In the search for the factors that can regulate the dWAT compartment, Donati et al. uncovered evidence that activation of epidermal Wnt/β-catenin signaling induced adipogenic differentiation in the dermis by secretion of ligands for the Bmp (Bmp2/Bmp6) and insulin (Igf2) signaling pathways [8]. Likewise, our recent study showed that epidermally expressed Foxn1 can participate in signaling pathways regulating DF adipogenic differentiation. Studies on mice with active Foxn1 (Foxn1^+/+^), partially inactivated Foxn1 (Foxn1^+/−^) or inactive Foxn1 (Foxn1^−/−^) revealed a gradual decrease in *Bmp2* and *Igf2* expression in the skin tissues as a function of gradual inactivation of Foxn1 [13]. To examine the impact of keratinocytes and the potential impact of epidermally expressed Foxn1 on DFs adipogenic differentiation, primary cultures of mouse keratinocytes were transduced with an adenoviral vector carrying Foxn1-GFP (Ad-Foxn1) or GFP alone (control; Ad-GFP) (Figure 4a) and then cocultured with DFs according to the presented scheme (Figure 4b).

Initially, using Trypan Blue exclusion and MTT assay, we determined that the presence of keratinocytes or adenoviral Foxn1/GFP transduction into keratinocytes under coculture conditions did not affect DFs number or viability (Figure 4c,d). Conversely, flow cytometry analysis of DFs revealed an increased number of viable DFs in coculture with keratinocytes transduced with Ad-Foxn1 in comparison to DFs under monolayer conditions (Figure 4e). Although not statistically significant, the population of necrotic cells increased under monolayer conditions (Figure 4e).

**Figure 5:**
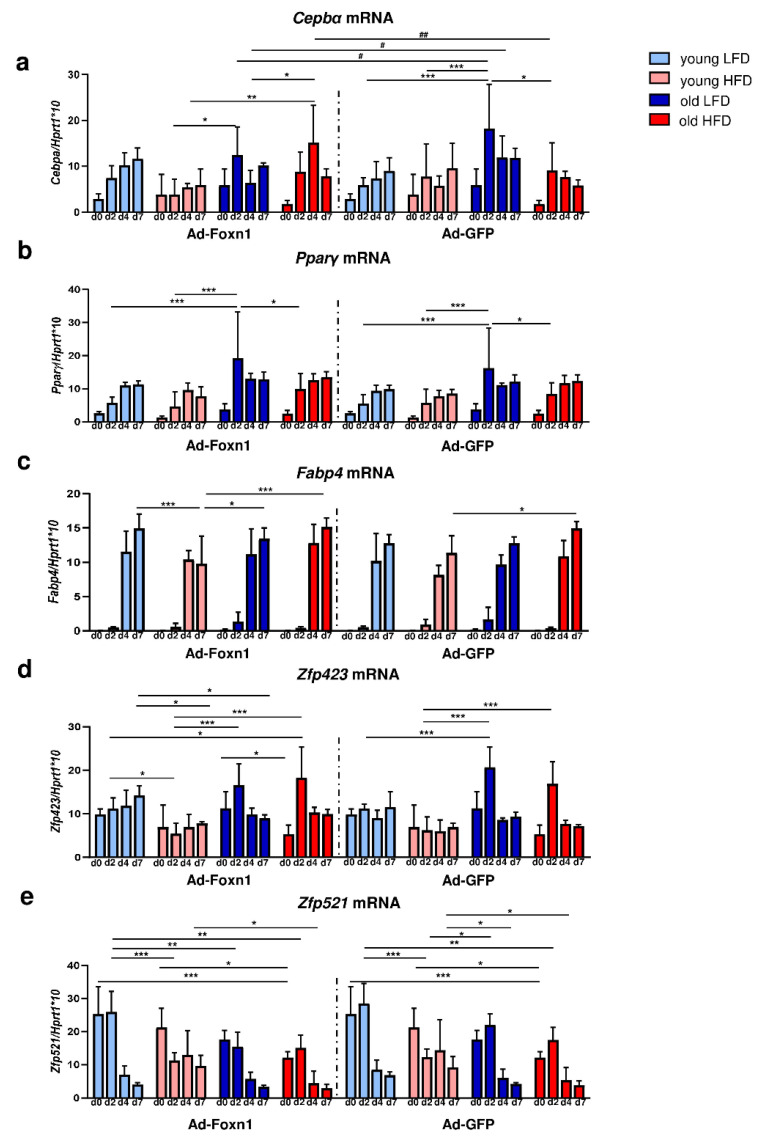
The effect of age, diet and epidermal Foxn1 signaling on DF adipogenic potential. Analysis of the expression of adipogenic-related genes in differentiated DFs in keratinocytes-DFs coculture conditions: (**a**) *Cepbα*, (**b**) *Pparγ*, (**c**) *Fabp4*, (**d**) *Zfp423*, (**e**) *Zfp521*. Values are the mean ± SD. Asterisks indicate significant differences within Ad-Foxn1 or Ad-GFP groups (* *p* < 0.05; ** *p* < 0.01; *** *p* < 0.001). Hashes indicate significant differences between Ad-Foxn1 vs. Ad-GFP groups (# *p* < 0.05; ## *p* < 0.01).

To evaluate potential changes, we examined the adipogenic capability of DFs isolated from the skin of young or old mice fed LFD or HFD and cocultured with keratinocytes transduced with Ad-Foxn1 or Ad-GFP (control). The expression levels of early (*Cebpα* and *Pparγ*) and late (*Fabp4*) markers of adipogenic differentiation and genes associated with the adipogenic commitment process (*Zfp423*, *Zfp521*) in DFs were analyzed (Figure 5).

The *Cebpα* mRNA expression increased during the course of adipogenic stimulation in DFs isolated from all experimental groups. However, the time of the peak of *Cebpα* expression differed between groups occurring at day 7 for young DFs while at day 2–4 for old DFs, regardless of keratinocyte adenoviral transduction (Figure 5a, Supplementary Table S2). Of note, Ad-Foxn1 overexpression in keratinocytes affected *Cebpα* expression in the cocultured DFs isolated from old mice fed LFD or HFD at days 2 and 4 of adipogenic process (Figure 5a).

The expression of *Pparγ* during DF adipogenic stimulation in the coculture model showed a gradual increase from day 2 until day 7 in all experimental groups (Figure 5b, Supplementary Table S3). We did not observe significant differences in the levels of *Pparγ* mRNA expression between DFs cocultured with keratinocytes transduced with Ad-Foxn1 or Ad-GFP (Figure 5b, Supplementary Table S3). As expected, a surge in the late marker of adipogenic differentiation, the expression of *Fabp4* mRNA, was observed at day 4 of adipogenic stimulation in both coculture conditions (Ad-Foxn1 or Ad-GFP) (Figure 5c, Supplementary Table S4). Similar to the *Cepbα* levels of expression, the lowest expression of *Fabp4* mRNA was observed for DFs isolated from young animals fed HFD, however, no statistical significance was found between coculture settings (Ad-Foxn1 vs. Ad-GFP).

Next, we analyzed the expression of the recently identified determinant of preadipocyte commitment, *Zfp423*, in differentiated DFs (Figure 5d). The mRNA levels of *Zfp423* in DFs isolated from young mice was relatively stable regardless of diet while displaying a sharp peak at day 2 of adipogenic stimulation in DFs isolated from old mice regardless of diet (Figure 5d, Supplementary Table S5). We also examined the expression levels of *Zfp521*, a negative regulator of adipogenesis, in DFs during adipogenic stimulation (Figure 5e). The sharp decrease in mRNA levels of *Zfp521* expression was observed at day 2 (DFs from young HFD mice) or after day 2 (all other groups of DFs) of adipogenesis (Figure 5e, Supplementary Table S6). Moreover, *Zfp521* expression decreased as a function of animal age (being the highest in young mice; Figure 5e).

### 2.5. Foxn1 in Keratinocytes Stimulates the Increase in Bmp2 and Igf2, Signal Transducers of the Pro-Adipogenic Pathway

**Figure 6:**
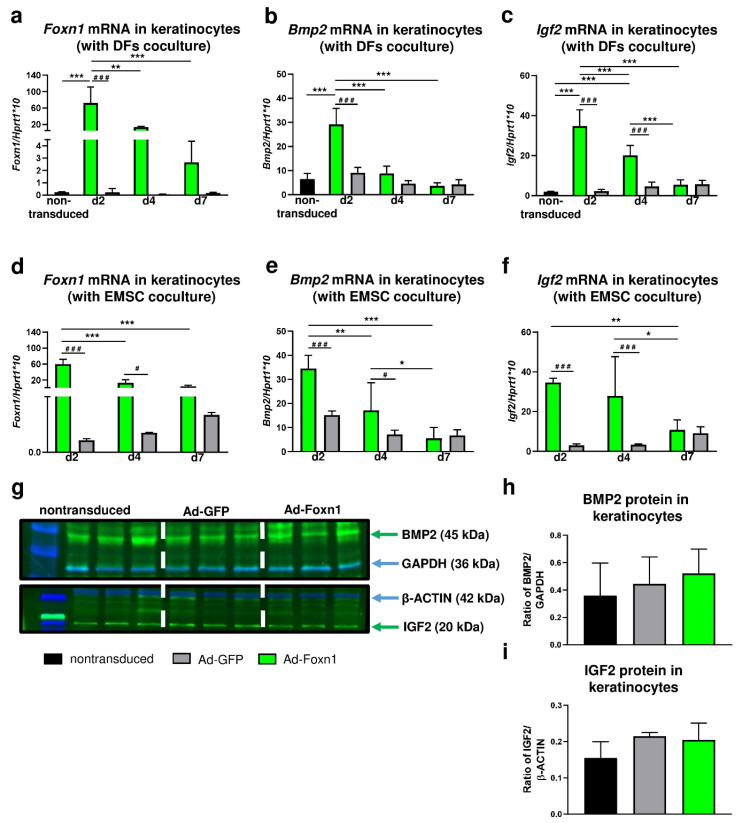
Overexpression of Foxn1 stimulates pro-adipogenic pathways Bmp2 and Igf2 in keratinocytes. (**a**) *Foxn1*, (**b**) *Bmp2* and (**c**) *Igf2* mRNA expression in keratinocytes cocultured with DFs. (**d**) *Foxn1*, (**e**) *Bmp2* and (**f**) *Igf2* mRNA expression in keratinocytes cocultured with EMSC. Western blot and densitometry analysis of BMP2 (**g**,**h**) and IGF2 (**g**,**i**) in keratinocytes nontransduced or transduced with Ad-Foxn1 or Ad-GFP. Values are the mean ± SD. Asterisks indicate significant differences among days of differentiation within keratinocytes transduced with Ad-Foxn1 or Ad-GFP (* *p* < 0.05; ** *p* < 0.01; *** *p* < 0.001). Hashes indicate significant differences between keratinocytes transduced with Ad-Foxn1 vs. Ad-GFP (# *p* < 0.05; ### *p* < 0.001).

The modest differences in the expression of adipogenic related genes in DFs cocultured with Ad-Foxn1 or Ad-GFP transduced keratinocytes motivated us to evaluate the effect of Foxn1 on the signal transducers of the pro-adipogenic pathway: Bmp2 and Igf2 in cocultured keratinocytes. Firstly, we examined *Foxn1* mRNA expression after adenovirus transduction (Figure 6a,d).

In experiments using keratinocytes-DFs (Figure 6a) or keratinocytes-EMSC (Figure 6d) cocultures, high levels of *Foxn1* mRNA were detected in keratinocytes transduced with Ad-Foxn1 regardless of coculture models. The highest expression of *Foxn1* in keratinocytes was observed at day 2 post-transduction that gradually decreased until day 7 (Figure 6a,d). Next, we analyzed the levels of mRNA expression for *Bmp2* (Figure 6b,e) and *Igf2* (Figure 6c,f) in keratinocytes. Interestingly, the expression pattern of both *Bmp2* and *Igf2* followed the *Foxn1* mRNA expression pattern in both coculture paradigms: keratinocytes-DFs (Figure 6a–c) or keratinocytes—EMSC (Figure 6d–f). The highest levels of *Bmp2* or *Igf2* were observed at day 2 post-Ad-Foxn1 transduction that gradually decreased to the levels of expression detected in nontransduced cells at day 7. The BMP2 and IGF2 protein abundance was measured by Western blot and densitometry in monolayer cultured keratinocytes that were transduced with: Ad-Foxn1, Ad-GFP or nontransduced (Figure 6g–i). Keratinocytes that were transduced with control (Ad-GFP) adenovirus noncarrying Foxn1 showed low and unchanging levels of *Bmp2* (Figure 6b,e) and *Igf2* (Figure 6c,f). Although the analysis revealed an increase in BMP2 (Figure 6g,h) and IGF2 (Figure 6g,i) protein levels upon Foxn1 stimulation/overexpression, the results were not statistically significant.

## 3. Discussion

In recent years, there has been an increasing interest in dermal white adipose tissue (dWAT), a newly recognized fat depot within the dermal skin compartment [1,15,26]. Due to the fact that dWAT is developmentally, biochemically and functionally different from other white adipose tissue/fat depots (i.e., subcutaneous, intraperitoneal) [2,26] it is essential to examine and understand its function and role in skin homeostasis. It has been well-established that dWAT is regulated in a manner that is distinct from other fat depots, displaying parallels to hair follicle cycling. During the active growth phase of the hair cycle (anagen), dWAT thickness increases while following the regression stage of the hair cycle (catagen), dWAT thickness decreases considerably [8,27,28]. Furthermore, the population of immature dWAT adipocytes plays a role in proper hair cycling by participation in induction of telogen to anagen transition [3].

In our study we examined how systematic/environmental changes, i.e., diet (LFD vs. HFD) and age (young vs. old), affect dWAT and its cellular component. We showed that (1) HFD during a period of 8 weeks stimulates dWAT expansion in the context of dWAT area extension in the skin, adipocyte diameter/size and percentage of extracted lipids, regardless of animals’ age (young vs. old mice) and (2) the increase in dWAT content due to HFD is achieved at least partially through hyperthrophy as indicated by the increase in adipocytes size and diameter. This data suggests that dWAT, similar to other fat depots, is regulated by diet as presented previously [29]. However, evidence from other fat depots, namely comparison of epididymal (eWAT) vs. inguinal (iWAT), revealed that the differences in fat tissue enlargement due to an obesogenic environment was achieved through hypertrophy (eWAT) and/or hyperplasia (iWAT) [30]. To shed light on changes related to age and/or diet, phenotypic characterization of DFs with adipogenic properties was performed. We distinguished three major skin cell populations: CD26^+^ (approx. 60%), CD26^+^/PDGFRα^+^ (approx. 20%) and PDGFRα^+^ (approx. 4%), the latter group associated with the predominant profibrotic nature of DFs. The data also showed that those populations were percentage stable and were not affected by animals’ age or diet. Further analyses revealed that the subpopulation of PDGFRα^+^/CD24^+^/Sca1^+^ DFs, previously recognized as adipocyte progenitors [17,18], decreased with animals’ age particularly in the group fed LFD, indicating a reduced capacity to differentiate into mature adipocytes. Interestingly, the detected decrease observed in the CD24^+^ cell population due to age seemed to be compensated by the increase in the percentage of Sca1^+^ in the old HFD mice. These results are in line with previous findings showing that the loss of adipocyte progenitor cells with aging may affect the wound healing process [31]. Further evidence of the reduced pool of adipocyte progenitor cells with age is provided by the analysis of the transcription factor *Zfp521* expression. Kang et al. identified Zfp521 as a potent repressor of adipogenesis in vivo and in vitro [21]. Our data indicate that expression of *Zfp521* decreased with animals’ age correlating with a decrease in the population of CD24^+^ cells. Another transcriptional regulator involved in preadipocyte determination is *Zfp423* [20]. The decrease observed in the expression of *Zfp423* of animals fed HFD may indicate that the pool of cells with adipogenic potential achieved adipogenic differentiation capacity whereas higher mRNA of *Zfp423* levels in DFs isolated from mice fed LFD may indicate that it still contains a population of DFs with adipogenic ability. However, analyses to evaluate whether changes in dWAT expansion involves adipocyte stem cell/progenitor recruitment and if advanced age of animals affects the pool of dWAT progenitors, will require further in vivo study, i.e., involving label-retaining cells (LRC) analysis [30]. Nevertheless, the present data broadens the complexity of dWAT regulation by showing that in addition to its local regulation by hair cycling, the systemic factors age and diet exhibit considerable impact.

Previous studies on subcutaneous adipose tissue have indicated that the size of the fat cell was associated with its secretory activity of adipokines and expression of pro- and anti-inflammatory factors [32,33]. Moreover, heterogeneity of adipocyte size affects subsequent alternations in lipid and energy metabolism. This is consistent with our results which showed that due to the obesogenic environment the total content of lipids in the skin increased which correlated with intradermal adipocyte size and the general content of dWAT in uninjured skin. Considering the secretory role of dWAT in the wound healing process [34], understanding mechanisms of age- and diet-dependent changes on lipid content in the skin remains an important issue for future research.

Schmidt and Horsley demonstrated that the population of adipocyte precursor cells and mature adipocytes is necessary for efficient skin wound repair [5]. The presence of intradermal adipocytes affects the function of DFs by stimulating their migration to the wound bed [5]. Authors also showed that adipocyte-conditioned medium applied to explants of tail skin enhanced dermal fibroblast migration but had no effect on DF proliferation [5]. Our present study based on an ex vivo model revealed that HFD accelerated dermal fibroblasts’ migratory abilities and increased content of dWAT in uninjured skin. Interestingly, migration of DFs was significantly enhanced in old animals fed HFD especially in contrast to old animals fed LFD. This may be an additional evidence of compensatory role of HFD on skin homeostasis in older animals. Our previous studies indicated that mice fed HFD had a thicker dWAT layer which may compensate for the loss in age-related thickness of the fibroblast-rich dermis and may have a beneficial effect on the skin wound healing process [14]. Although several studies specify caloric restriction as a favorable dietary intervention which can delay age-related changes in the structure and metabolism of the skin [19,35], the physiological increase in dWAT with age may be beneficial as our previous [14] and present study indicate.

Despite increasing interest in the role of dWAT in skin physiology, the current knowledge considering dWAT homeostasis and molecular pathways is limited. Donati et al. demonstrated that the epidermal Wnt/β-catenin signaling pathway is a key initiator of the signal transduction cascade that stimulates the process of adipogenesis in the skin [8]. Furthermore, the supplementation of adipogenic media with Bmp2, Bmp6 and Igf2 proteins also stimulated the process of adipogenesis suggesting that the Wnt/β-catenin pathway promotes adipocyte differentiation by stimulating secretion of ligands for the Bmp (Bmp2/Bmp6) and insulin (Igf2) signaling pathways [8]. Our recently published data revealed that the transcription factor Foxn1, whose expression is limited to epithelial cells of the skin, may affect skin adiposity. A series of in vivo experiments using transgenic Foxn1::Egfp mice (Foxn1^+/−^) and B6 mice (Foxn1^+/+^) demonstrated that young and old Foxn1^+/−^ mice fed HFD gained significantly less body weight than their male B6 counterparts, indicating the resistance of heterozygous Foxn1^+/−^ mice to diet-induced obesity [13]. Moreover, expression of adipogenic-related genes (*Cepbα*, *Pparγ*, *Fabp4*, *leptin*) was abrogated in injured and intact skin of Foxn1^+/−^ mice. Interestingly, we observed that with gradual decrease of Foxn1 activation in the skin of Foxn1^+/+^, Foxn1^+/−^ and Foxn1^−/−^ mice, expression of *Bmp2* and *Igf2* decreased correspondingly [13]. In the present study, we analyzed the expression of *Bmp2* and *Igf2* in keratinocytes after transduction with Foxn1-GFP-expressing (Ad-Foxn1) or control (Ad-GFP) adenoviruses. In vitro experiments revealed increased expression of *Bmp2* and *Igf2* with overexpression of Foxn1 confirming the relationship between the Foxn1 activity and expression of adipogenesis-promoting genes.

Considering participation of Foxn1 in the Bmp2 and Igf2 signaling pathways [13] and the previously described double paracrine mechanism between keratinocytes and fibroblasts [24], we analyzed DF adipogenic differentiation in a coculture system with Ad-Foxn1- vs. Ad-GFP- (control) transduced keratinocytes. The analysis of adipogenic genes (*Cebpα*, *Pparγ*, *Fabp4*) in differentiating DFs showed differences among the groups due to age and diet, especially for markers characteristic for the initial stage of the adipogenesis process (*Cebpα* and *Pparγ*). Surprisingly, the highest expression of adipogenic genes was observed in DFs isolated from old animals fed LFD. The changes detected in aged DFs may possibly cause the loss of functional identity and the acquisition of pro-adipogenic features. Salzer et al. revealed that during aging DFs displayed reduction in extracellular matrix (ECM) genes i.e., collagens and glycosaminogycans with simultaneously higher expression of markers of the immune response and the adipogenesis process, including lipogenesis and fat cell differentiation [19].

The differences in the process of DF differentiation due to Ad-Foxn1 presence in keratinocytes has been exclusively detected for *Cebpα* expression (see Figure 5a). This mild effect of Foxn1 on DF adipogenesis in our present in vitro study may indicate that this model is not sufficient to mirror the in vivo conditions. However, considering the robust expression of Foxn1 in hair follicles and interfollicular epidermis [9,36,37], changes in the adiposity in Foxn1^+/+^, Foxn1^+/−^ and Foxn1^−/−^ mice [13], the stimulatory effect of Foxn1 in keratinocytes on the release of adipogenic signaling factors Bmp2, Igf2 (present data) and the decline in the skin Foxn1 expression in very old mice [13], it strongly suggests a role of Foxn1 in dWAT physiology.

Collectively, our data indicate that diet and age regulate skin homeostasis influencing dWAT morphology and functionality. DFs display the adipogenic potential of differentiation that is comparable to that of SVF of fat depot and EMSC models. Furthermore, we showed that age and diet affect the pool of DFs with adipogenic properties. We also propose that the transcription factor Foxn1 expressed in epidermis has a stimulatory effect on the adipogenic signaling of Bmp2 and Igf2.

## 4. Materials and Methods

### 4.1. Animals

The studies were performed on B6 mice. All mice were obtained from colonies established at the animal facility of the Institute of Animal Reproduction and Food Research, The Polish Academy of Sciences. B6 mice were originally purchased from the Jackson Laboratory (Bar Harbor, ME, USA). Animals were bred and housed in a temperature- and humidity-controlled room (22 °C ± 2 °C and 35–65%, respectively) with a 12-h light/12-h dark cycle at the Institute of Animal Reproduction and Food Research, Polish Academy of Sciences, Olsztyn, Poland.

Young (2 months) and old (18 months old) mice were divided into two experimental groups fed (1) a low-fat diet (11% kcal from fat, PicoLab Rodent Diet 20 5053) and (2) a high-fat diet (58% kcal from fat, TestDiet AIN-76A; LabDiet) for 8 weeks. Skin samples from the back of mice were collected postmortem for cell isolation, ex vivo explants or histological assays.

The experimental animal procedures performed in these studies were approved 29 April 2015 by the Ethics Committee of The University of Warmia and Mazury (Olsztyn, Poland), No. 22/2015.

### 4.2. Histology

Immunohistochemical detection of perilipin (1:200, ab3526, Abcam Cambridge, MA, USA) was performed on formalin-fixed samples that were processed, embedded in paraffin blocks and sectioned at 5 μm. Heat-induced antigen retrieval followed by ABC detection (Vectastain ABC kit, Vector Laboratories, Inc., Burlingame, CA, USA) was performed using the protocols described previously [11]. Peroxidase activity was revealed using 3.3-diaminobenzidine (Sigma-Aldrich, St. Louis, MO, USA) as a substrate. Slides were counterstained with hematoxylin and visualized using an Olympus microscope (BX43, Olympus, Tokyo, Japan).

Measurements of percentage of the adipocyte area in the skin, and diameter and size of intradermal adipocytes were performed using ImageJ software version v1.52a (National Institutes of Health, Bethesda, MD, USA) (number of mice *n* = 5 per group).

### 4.3. Lipid Extraction

Lipid extraction according to the Folch method with modifications described previously [38,39] was performed on uninjured skin collected from young, old, fed LFD or HFD mice. Briefly, tissues were weighed and homogenized with a 2:1 mixture of chloroform–methanol (4 mL of mixture per 0.05 g of tissue) (*n* = 4 per group). After homogenization, mixtures were centrifuged at 15,000× *g* for 10 min and supernatants were transferred to the new tubes with addition of 0.8 mL of distilled water, vortexed and centrifuged for 15 min (2500× *g*). Upper phases were removed and lower phases with lipid content were evaporated under a nitrogen stream at 37 °C for 35 min. Percentage of lipid content was calculated by dividing weight of lipids after evaporation by weight of tissue and multiplying by 100.

### 4.4. Ex Vivo DF Migration Assay

Skin explants collected postmortem from young or old B6 mice fed LFD or HFD were cut into small (1–2 mm^2^) samples, seeded in 60-mm Petri dishes (10 samples per plate) and cultured in DMEM/F-12 medium (Sigma-Aldrich, St. Louis, MO, USA) supplemented with 15% of fetal bovine serum (FBS; Life Technologies, Thermo Fisher Scientific, Waltham, MA, USA) and antibiotics (Penicillin/Streptomycin, Sigma-Aldrich, St. Louis, MO, USA). Every second day media was removed and replaced with a fresh equivalent volume. Dermal fibroblast migration was photo documented daily and evaluated by the DF area of outgrowth from skin explant using ImageJ (National Institutes of Health, Bethesda, MD, USA) similar to the previously described method for keratinocytes migration [40].

### 4.5. Flow Cytometry Analysis

EMSC and DFs isolated from young or old B6 mice fed LFD or HFD (*n* = 3–5) were characterized by expression of cell surface markers using flow cytometry analysis. Briefly, cells (*p* = 1) seeded in a 60-mm culture dish were cultured in DMEM/F12 culture medium (Sigma-Aldrich, St. Louis, MO, USA) supplemented with 15% fetal bovine serum (FBS, Life Technologies, Thermo Fisher Scientific, Waltham, MA, USA) with antibiotics (Penicilin/Streptomycin, Sigma-Aldrich, St. Louis, MO, USA). Confluent cells were washed with PBS, trypsinized and counted (Countess, Life Technology, Thermo Fisher Scientific, Waltham, MA, USA). For the analysis, approximately 0.6–1.5 × 10^6^ cells from each set of cells were incubated for 30 min with fluorochrome-labeled antibodies to specific surface antigens or their isotype controls (Supplementary Table S7) and fixed with 1% PFA for 20 min. Cells were analyzed the same day using a BD LSRFortessa cell analyzer flow cytometer and BD FACSDiva TM v6.2 software (Becton Dickinson, Franklin Lakes, NJ, USA).

### 4.6. Cell Isolation and Culture

Skin samples were mechanically processed and digested in collagenase type 1 (3.68 mg/mL; Sigma-Aldrich, St. Louis, MO, USA) for 80 min at 37 °C in an incubator shaker. Isolates were filtered with a cell strainer with a pore diameter of 100 μm and centrifuged for 5 min at 1300 rpm at room temperature. Cells were plated in 60- or 100-mm Petri dishes in DMEM/F-12 medium (Sigma-Aldrich, St. Louis, MO, USA) with 15% fetal bovine serum (FBS, Life Technologies, Thermo Fisher Scientific, Waltham, MA, USA) and gentamicin/amphotericin solution (Life Technologies, Thermo Fisher Scientific, Waltham, MA, USA). Confluent fibroblasts were trypsinized (0.05%; Sigma-Aldrich, St. Louis, MO, USA) and frozen for further experiments. Ear mesenchymal stem cells (EMSC) and stromal vascular fraction (SVF) of inguinal fat depot were isolated and cultured as described before [23].

Skin tissues isolated from newborn B6 mice were incubated overnight in dispase solution (6 U/mL; Life Technologies) at 4 °C to separate epidermis from dermis. Keratinocytes were isolated from separated epidermis by a series of trypsin digestions as previously described [41]. Cells were counted and seeded in cell culture inserts with a 0.4-μm pore size membrane (0.5 × 10^6^ per insert). Keratinocytes were seeded in DMEM/F-12 medium (Sigma-Aldrich, St. Louis, MO, USA) supplemented with 10% FBS (Life Technologies, Thermo Fisher Scientific, Waltham, MA, USA), 0.2% Primocin (InvivoGen, San Diego, CA, USA) and 120 μM β-mercaptoethanol (Sigma-Aldrich, St. Louis, MO, USA). After 24 h of culture, seeding medium was switched to keratinocyte-specific medium (CellnTec, Bern, Switzerland).

### 4.7. Keratinocyte Transduction with Adenoviruses

Keratinocyte transduction was performed accordingly to a previously described protocol [41]. Briefly, keratinocytes were seeded in inserts (Corning^®^ BioCoat™ Control Inserts with a 0.4-µm PET Membrane; Corning, Tewksbury, MA, USA) were transduced with Foxn1-GFP-expressing (Ad-Foxn1) or control (Ad-GFP) adenoviruses at 70% of confluency. After 4 h, 1 mL of CnT Basal Medium with supplements A, B, C (CELLnTEC, Bern, Switzerland) were added per each insert and coculture of keratinocytes-DFs or keratinocytes-EMSC models were set up (day 0). The keratinocytes medium was changed every 2 days of culture.

### 4.8. DF/SVF/EMSC Adipogenic Differentiation

Adipogenic differentiation was performed as previously described [42]. Cells (DFs, SVFs or EMSC) were seeded in a 6-well plate and maintained in DMEM/F12 supplemented with 15% FBS and penicillin/streptomycin (Life Technologies, Thermo Fisher Scientific, Waltham, MA, USA), until confluence (day 0). The adipogenic differentiation process in DFs, SVFs, or EMSC was induced by changing media to Adipogenic Medium I containing DMEM/F12, 5% FBS, 1% Penicillin/Streptomycin, 0.5 mM isobutylmethylxanthine (IBMX, Sigma-Aldrich, St. Louis, MO, USA), 1.7 μM insulin (Sigma-Aldrich, St. Louis, MO, USA) and 1 μM dexamethasone (Sigma-Aldrich, St. Louis, MO, USA). At day 2 of differentiation, the medium was replaced by Adipogenic Medium II containing DMEM/F12 supplemented with 5% FBS, 1% Penicillin/Streptomycin, 17 nM insulin and 5 μM rosiglitazone (Sigma-Aldrich, St. Louis, MO, USA). Cells (DFs/EMSC/keratinocytes) were harvested at indicated time points: day 0, 2, 4, 7 for RNA isolation.

### 4.9. Oil Red O Staining

DFs or SVF cells were washed with PBS, fixed in 10% buffered formalin for 1 h and stained with a 60% solution of Oil Red O (Sigma-Aldrich, St. Louis, MO, USA) for 10 min as described previously [43]. Cells were washed five times with water and visualized under a microscope (IX51, Olympus, Tokyo, Japan) equipped with an Olympus digital camera (XC50, Olympus, Tokyo, Japan). Lipid accumulation was measured by extracting Oil Red O from dye-retaining cells with isopropanol, and determining absorbance at 500 nm.

### 4.10. Cell Metabolic Activity Measurement and Viability Assay

DFs isolated from young LFD animals were cultured in monolayer condition or in coculture with keratinocytes nontransduced or transduced with Ad-Foxn1 or Ad-GFP (*n* = 5 per condition).

DF cell metabolic activity was measured using MTT colorimetric method as described previously [43]. Briefly, after 24 h and 48 h, sterile MTT solution (3-[4,5-dimethylthiazol- 2-yl]-2,5-diphenyltetrazolium bromide; Sigma-Aldrich, St. Louis, MO, USA) was added to each well and incubated for 4 h. Next, the medium was removed and formazan crystals were dissolved in DMSO for 1 h. Absorbance was measured at 570 nm using a microplate reader (ASYS Hitech GmbH, UVM340, Biogenet; Biochrom Ltd., Cambridge, UK) and MicroWin 2000 software version V4.0 for Windows XP (Siemens, Monachium, Germany).

For the viability assay after 24 h of culture, cells were trypsinized, counted using the trypan blue exclusion method and resuspended in Annexin Binding Buffer (Life Technologies, Thermo Fisher Scientific, Waltham, MA, USA) with addition of Annexin V Alexa Fluor 350 Conjugate (Life Technologies) and propidium iodide (Life Technologies). Samples were analyzed using flow cytometry with BD LSRFortessa and BD FACSDiva TM v6.2 software (Becton Dickinson, Franklin Lakes, NJ, USA).

### 4.11. RNA Isolation and Quantitative Real-Time Reverse Transcriptase PCR

Real-time RT-qPCR was performed as described previously [11]. Total RNA was isolated using the TRIzol Reagent (Invitrogen, Thermo Fisher Scientific, Waltham, MA, USA). RNA (500 ng) was reverse transcribed with the High-Capacity cDNA Reverse Transcription Kit with RNase Inhibitor (Applied Biosystems, Thermo Fisher Scientific) according to the manufacturer’s instructions. The mRNA levels of adipogenic-related genes were measured with Single Tube TaqMan Gene Expression Assays (Life Technologies, Thermo Fisher Scientific) (Supplementary Table S8). The levels of gene expression were quantified relative to the level of *Hprt1*.

### 4.12. Protein Isolation and Western Blot Analysis

Cell lysate collected from nontransduced, transduced with Ad-Foxn1 or Ad-GFP keratinocytes were prepared in 200 μL RIPA buffer containing protease inhibitor cocktail (PhosStop, Roche; Protease Inhibitor, Sigma-Aldrich, St. Louis, MO, USA). Thirty milligrams of protein per sample separated on 12% SDS/PAGE gels and transferred onto polyvinylidene difluoride membranes (Merck Millipore, Burlington, MA, USA) were incubated with anti-BMP2 (1:1000, ab14933, Abcam, Cambridge, MA, USA), anti-IGF2 (1:800, GTX 129110, GeneTex, Irvine, CA, USA), anti-GAPDH (1:1500, ab 9484, Abcam, Cambridge, MA, USA) and anti-Actb (1:1000, ab 8226, Abcam, Cambridge, MA, USA) antibodies followed by fluorescent secondary antibodies (Alexa Fluor 680, 1:10,000, goat anti-mouse, A10038, Life Technologies, Thermo Fisher Scientific and Irdye 800, 1:10,000, 611-132-122, Rockland, Limerick, PA, USA). Bands were visualized using the ChemiDoc™ Imaging System (Bio-Rad Laboratories, Inc., Hercules, CA, USA). Densitometric analysis was performed using Image Lab™ Software version 6.0 (Bio-Rad Laboratories, Inc.) (*n* = 3 samples per experimental group).

### 4.13. Statistical Analysis

Linear models were used in order to analyze the impact of different variables on parameters, in which interaction between these covariates was taken into account as well. Based on such models, lsmeans were calculated (least-squared means—means calculated based on model’s coefficients) and compared. For each model, the adjusted Rsquared is provided as the measure of goodness-of-fit. The significance level was set to 0.05. Calculations were performed in R (ver. 3.5.3) using packages: emmeans (ver. 1.4.1) and tidyverse (ver. 1.2.1).

Statistical analysis of DF flow cytometric characteristics, EMSC and DF adipogenic differentiation was performed with GraphPad Prism, Version 6.0 (GraphPad Software, La Jolla, CA, USA). The data were checked for normality using the Shapiro–Wilk test. One-way analysis of variance with posthoc Tukey’s test or two-way analysis of variance were used. Data are expressed as mean ± standard deviation (SD). A value of *p* < 0.05 was considered statistically significant.

## Supplementary Materials

Supplementary materials can be found at https://www.mdpi.com/1422-0067/21/23/8955/s1.
