# Supplementary material for: Age, Diet and Epidermal Signaling Modulate Dermal Fibroblasts’ Adipogenic Potential
Source: Int J Mol Sci. 2020 Nov 25;21(23):8955. doi: 10.3390/ijms21238955 (PMC7728337; doi:10.3390/ijms21238955)
Supplement: Supplementary file 1 [file ijms-21-08955-s001.pdf]

Supplementary Materials

Age, Diet and Epidermal Signaling Modulate Dermal Fibroblasts' Adipogenic Potential

Table S1. Dermal fibroblasts migration—comparisons by day.

GROUP	DAY	LSMEAN	LOWER.CL	UPPER.CL	SE	DF	2D	3D	4D	6D	7D
**YOUNG LFD**	1d	0.00	−9.16	9.16	4.62	101.00	1.000	0.987		<0.001	<0.001
**YOUNG LFD**	2d	−0.00	−9.16	9.16	4.62	101.00		0.987		<0.001	<0.001
**YOUNG LFD**	3d	4.25	−4.92	13.41	4.62	101.00				0.004	<0.001
**YOUNG LFD**	4d	NA	NA	NA	NA	NA					
**YOUNG LFD**	6d	28.52	19.36	37.68	4.62	101.00					0.006
**YOUNG LFD**	7d	54.65	43.70	65.60	5.52	101.00					
**YOUNG HFD**	1d	0.00	−14.49	14.49	7.30	101.00	1.000	0.728	<0.001		
**YOUNG HFD**	2d	1.58	−12.91	16.07	7.30	101.00		0.813	<0.001		
**YOUNG HFD**	3d	14.45	−0.04	28.93	7.30	101.00			0.004		
**YOUNG HFD**	4d	53.26	38.77	67.75	7.30	101.00					
**YOUNG HFD**	6d	NA	NA	NA	NA	NA					
**YOUNG HFD**	7d	NA	NA	NA	NA	NA					
**OLD LFD**	1d	0.00	−12.96	12.96	6.53	101.00	1.000	1.000		1.000	0.999
**OLD LFD**	2d	0.00	−12.96	12.96	6.53	101.00		1.000		1.000	0.999
**OLD LFD**	3d	1.25	−11.70	14.21	6.53	101.00				1.000	1.000
**OLD LFD**	4d	NA	NA	NA	NA	NA					
**OLD LFD**	6d	1.90	−11.06	14.86	6.53	101.00					1.000
**OLD LFD**	7d	3.10	−9.86	16.06	6.53	101.00					
**OLD HFD**	1d	16.18	7.02	25.34	4.62	101.00	0.001	<0.001			
**OLD HFD**	2d	42.91	33.75	52.08	4.62	101.00		0.025			
**OLD HFD**	3d	63.54	54.38	72.70	4.62	101.00					
**OLD HFD**	4d	NA	NA	NA	NA	NA					
**OLD HFD**	6d	NA	NA	NA	NA	NA					
**OLD HFD**	7D	NA	NA	NA	NA	NA					

Table S2. *Cepbα* mRNA expression in DFs with keratinocytes coculture—comparisons by day.

	GROUP	TIME	LSMEAN	LOWER.CL	UPPER.CL	SE	DF	D2	D4	D7
**2M LFD**	Ad-Foxn1	d0	2.85	−1.06	6.77	1.97	104.00	0.357	0.033	0.007
**2M LFD**	Ad-Foxn1	d2	7.45	3.53	11.36	1.97	104.00		0.727	0.403
**2M LFD**	Ad-Foxn1	d4	10.20	6.70	13.70	1.77	104.00			0.944
**2M LFD**	Ad-Foxn1	d7	11.59	8.09	15.09	1.77	104.00			
**2M LFD**	Ad-GFP	d0	2.85	−1.06	6.77	1.97	104.00	0.704	0.370	0.129
**2M LFD**	Ad-GFP	d2	5.86	1.95	9.77	1.97	104.00		0.947	0.675
**2M LFD**	Ad-GFP	d4	7.39	3.47	11.30	1.97	104.00			0.938
**2M LFD**	Ad-GFP	d7	9.00	5.09	12.92	1.97	104.00			
**2M HFD**	Ad-Foxn1	d0	3.85	0.35	7.35	1.77	104.00	1.000	0.936	0.892
**2M HFD**	Ad-Foxn1	d2	3.81	−0.71	8.33	2.28	104.00		0.953	0.916
**2M HFD**	Ad-Foxn1	d4	5.40	1.48	9.31	1.97	104.00			0.998
**2M HFD**	Ad-Foxn1	d7	5.90	1.38	10.42	2.28	104.00			
**2M HFD**	Ad-GFP	d0	3.85	0.35	7.35	1.77	104.00	0.455	0.893	0.142
**2M HFD**	Ad-GFP	d2	7.76	3.85	11.68	1.97	104.00		0.885	0.917
**2M HFD**	Ad-GFP	d4	5.73	1.81	9.64	1.97	104.00			0.519
**2M HFD**	Ad-GFP	d7	9.56	5.65	13.48	1.97	104.00			
**18M LFD**	Ad-Foxn1	d0	5.83	1.92	9.75	1.97	104.00	0.093	0.998	0.409
**18M LFD**	Ad-Foxn1	d2	12.40	8.49	16.32	1.97	104.00		0.139	0.855
**18M LFD**	Ad-Foxn1	d4	6.35	2.44	10.27	1.97	104.00			0.521
**18M LFD**	Ad-Foxn1	d7	10.17	6.26	14.09	1.97	104.00			
**18M LFD**	Ad-GFP	d0	5.83	1.92	9.75	1.97	104.00	<0.001	0.136	0.114
**18M LFD**	Ad-GFP	d2	18.20	14.70	21.70	1.77	104.00		0.089	0.058
**18M LFD**	Ad-GFP	d4	11.92	8.00	15.83	1.97	104.00			1.000
**18M LFD**	Ad-GFP	d7	11.82	8.32	15.32	1.77	104.00			
**18M HFD**	Ad-Foxn1	d0	1.75	−1.21	4.71	1.49	104.00	0.052	<0.001	0.076
**18M HFD**	Ad-Foxn1	d2	8.83	4.31	13.35	2.28	104.00		0.164	0.986
**18M HFD**	Ad-Foxn1	d4	15.13	11.21	19.04	1.97	104.00			0.048
**18M HFD**	Ad-Foxn1	d7	7.79	3.88	11.71	1.97	104.00			
**18M HFD**	Ad-GFP	d0	1.75	−1.21	4.71	1.49	104.00	0.042	0.087	0.365
**18M HFD**	Ad-GFP	d2	9.06	4.53	13.58	2.28	104.00		0.966	0.701
**18M HFD**	Ad-GFP	d4	7.65	3.73	11.56	1.97	104.00			0.910
**18M HFD**	AD-GFP	D7	5.79	1.88	9.71	1.97	104.00			

Table S3. *Pparγ* mRNA expression in DFs with keratinocytes coculture—comparisons by day.

	GROUP	TIME	LSMEAN	LOWER.CL	UPPER.CL	SE	DF	D2	D4	D7
**2M LFD**	Ad-Foxn1	d0	2.54	−1.30	6.38	1.94	104.00	0.658	0.008	0.006
**2M LFD**	Ad-Foxn1	d2	5.69	1.85	9.53	1.94	104.00		0.180	0.139
**2M LFD**	Ad-Foxn1	d4	10.99	7.55	14.42	1.73	104.00			0.999
**2M LFD**	Ad-Foxn1	d7	11.32	7.89	14.76	1.73	104.00			
**2M LFD**	Ad-GFP	d0	2.54	−1.30	6.38	1.94	104.00	0.699	0.067	0.045
**2M LFD**	Ad-GFP	d2	5.51	1.67	9.35	1.94	104.00		0.499	0.400
**2M LFD**	Ad-GFP	d4	9.36	5.52	13.20	1.94	104.00			0.998
**2M LFD**	Ad-GFP	d7	9.81	5.97	13.65	1.94	104.00			
**2M HFD**	Ad-Foxn1	d0	1.24	−2.19	4.68	1.73	104.00	0.637	0.010	0.102
**2M HFD**	Ad-Foxn1	d2	4.59	0.16	9.03	2.24	104.00		0.338	0.746
**2M HFD**	Ad-Foxn1	d4	9.57	5.73	13.41	1.94	104.00			0.930
**2M HFD**	Ad-Foxn1	d7	7.78	3.34	12.21	2.24	104.00			
**2M HFD**	Ad-GFP	d0	1.24	−2.19	4.68	1.73	104.00	0.311	0.063	0.031
**2M HFD**	Ad-GFP	d2	5.75	1.91	9.59	1.94	104.00		0.879	0.749
**2M HFD**	Ad-GFP	d4	7.79	3.95	11.63	1.94	104.00			0.994
**2M HFD**	Ad-GFP	d7	8.49	4.66	12.33	1.94	104.00			
**18M LFD**	Ad-Foxn1	d0	3.74	−0.10	7.58	1.94	104.00	<0.001	0.005	0.006
**18M LFD**	Ad-Foxn1	d2	19.19	15.35	23.03	1.94	104.00		0.116	0.104
**18M LFD**	Ad-Foxn1	d4	13.02	9.18	16.86	1.94	104.00			1.000
**18M LFD**	Ad-Foxn1	d7	12.88	9.04	16.72	1.94	104.00			
**18M LFD**	Ad-GFP	d0	3.74	−0.10	7.58	1.94	104.00	<0.001	0.042	0.009
**18M LFD**	Ad-GFP	d2	16.21	12.78	19.65	1.73	104.00		0.205	0.355
**18M LFD**	Ad-GFP	d4	11.08	7.24	14.92	1.94	104.00			0.975
**18M LFD**	Ad-GFP	d7	12.17	8.73	15.60	1.73	104.00			
**18M HFD**	Ad-Foxn1	d0	2.47	−0.44	5.37	1.46	104.00	0.033	<0.001	<0.001
**18M HFD**	Ad-Foxn1	d2	9.88	5.44	14.31	2.24	104.00		0.801	0.615
**18M HFD**	Ad-Foxn1	d4	12.56	8.72	16.40	1.94	104.00			0.987
**18M HFD**	Ad-Foxn1	d7	13.49	9.65	17.33	1.94	104.00			
**18M HFD**	Ad-GFP	d0	2.47	−0.44	5.37	1.46	104.00	0.118	0.001	<0.001
**18M HFD**	Ad-GFP	d2	8.47	4.03	12.90	2.24	104.00		0.695	0.561
**18M HFD**	Ad-GFP	d4	11.70	7.86	15.54	1.94	104.00			0.996
**18M HFD**	AD-GFP	D7	12.33	8.49	16.17	1.94	104.00			

Table S4. *Fabp4*mRNA expression in DFs with keratinocytes coculture—comparisons by day.

	GROUP	TIME	LSMEAN	LOWER.CL	UPPER.CL	SE	DF	D2	D4	D7
**2M LFD**	Ad-Foxn1	d0	0.06	−1.61	1.73	0.84	104.00	0.985	<0.001	<0.001
**2M LFD**	Ad-Foxn1	d2	0.48	−1.18	2.15	0.84	104.00		<0.001	<0.001
**2M LFD**	Ad-Foxn1	d4	11.52	10.03	13.01	0.75	104.00			0.010
**2M LFD**	Ad-Foxn1	d7	14.93	13.44	16.42	0.75	104.00			
**2M LFD**	Ad-GFP	d0	0.06	−1.61	1.73	0.84	104.00	0.980	<0.001	<0.001
**2M LFD**	Ad-GFP	d2	0.52	−1.14	2.19	0.84	104.00		<0.001	<0.001
**2M LFD**	Ad-GFP	d4	10.17	8.51	11.84	0.84	104.00			0.134
**2M LFD**	Ad-GFP	d7	12.77	11.11	14.44	0.84	104.00			
**2M HFD**	Ad-Foxn1	d0	0.05	−1.44	1.54	0.75	104.00	0.978	<0.001	<0.001
**2M HFD**	Ad-Foxn1	d2	0.54	−1.38	2.47	0.97	104.00		<0.001	<0.001
**2M HFD**	Ad-Foxn1	d4	10.40	8.73	12.06	0.84	104.00			0.963
**2M HFD**	Ad-Foxn1	d7	9.78	7.85	11.70	0.97	104.00			
**2M HFD**	Ad-GFP	d0	0.05	-1.44	1.54	0.75	104.00	0.880	<0.001	<0.001
**2M HFD**	Ad-GFP	d2	0.88	-0.78	2.55	0.84	104.00		<0.001	<0.001
**2M HFD**	Ad-GFP	d4	8.16	6.49	9.82	0.84	104.00			0.038
**2M HFD**	Ad-GFP	d7	11.38	9.72	13.05	0.84	104.00			
**18M LFD**	Ad-Foxn1	d0	0.18	−1.49	1.84	0.84	104.00	0.749	<0.001	<0.001
**18M LFD**	Ad-Foxn1	d2	1.37	−0.30	3.03	0.84	104.00		<0.001	<0.001
**18M LFD**	Ad-Foxn1	d4	11.17	9.50	12.83	0.84	104.00			0.233
**18M LFD**	Ad-Foxn1	d7	13.43	11.76	15.09	0.84	104.00			
**18M LFD**	Ad-GFP	d0	0.18	−1.49	1.84	0.84	104.00	0.560	<0.001	<0.001
**18M LFD**	Ad-GFP	d2	1.65	0.16	3.14	0.75	104.00		<0.001	<0.001
**18M LFD**	Ad-GFP	d4	9.64	7.98	11.31	0.84	104.00			0.032
**18M LFD**	Ad-GFP	d7	12.78	11.29	14.27	0.75	104.00			
**18M HFD**	Ad-Foxn1	d0	0.04	−1.22	1.30	0.63	104.00	0.990	<0.001	<0.001
**18M HFD**	Ad-Foxn1	d2	0.39	−1.53	2.32	0.97	104.00		<0.001	<0.001
**18M HFD**	Ad-Foxn1	d4	12.82	11.15	14.48	0.84	104.00			0.220
**18M HFD**	Ad-Foxn1	d7	15.12	13.45	16.78	0.84	104.00			
**18M HFD**	Ad-GFP	d0	0.04	−1.22	1.30	0.63	104.00	0.992	<0.001	<0.001
**18M HFD**	Ad-GFP	d2	0.37	−1.55	2.29	0.97	104.00		<0.001	<0.001
**18M HFD**	Ad-GFP	d4	10.85	9.19	12.52	0.84	104.00			0.005
**18M HFD**	AD-GFP	D7	14.91	13.24	16.57	0.84	104.00			

Table S5. *Zfp423* mRNA expression in DFs with keratinocytes coculture—comparisons by day.

METHOD	GROUP	TIME	LSMEAN	LOWER.CL	UPPER.CL	SE	DF	D2	D4	D7
**AD-FOXN1**	2m LFD	d0	9.84	6.88	12.80	1.49	97.00	0.917	0.754	0.139
**AD-FOXN1**	2m LFD	d2	11.20	8.24	14.16	1.49	97.00		0.989	0.447
**AD-FOXN1**	2m LFD	d4	11.83	9.18	14.47	1.33	97.00			0.598
**AD-FOXN1**	2m LFD	d7	14.18	11.53	16.83	1.33	97.00			
**AD-FOXN1**	2m HFD	d0	6.89	4.25	9.54	1.33	97.00	0.877	1.000	0.979
**AD-FOXN1**	2m HFD	d2	5.40	2.44	8.35	1.49	97.00		0.890	0.729
**AD-FOXN1**	2m HFD	d4	6.91	3.95	9.87	1.49	97.00			0.982
**AD-FOXN1**	2m HFD	d7	7.75	4.34	11.17	1.72	97.00			
**AD-FOXN1**	18m LFD	d0	11.22	8.26	14.18	1.49	97.00	0.063	0.911	0.708
**AD-FOXN1**	18m LFD	d2	16.53	13.58	19.49	1.49	97.00		0.010	0.003
**AD-FOXN1**	18m LFD	d4	9.82	6.86	12.78	1.49	97.00			0.977
**AD-FOXN1**	18m LFD	d7	8.96	6.00	11.92	1.49	97.00			
**AD-FOXN1**	18m HFD	d0	5.33	2.38	8.29	1.49	97.00	<0.001	0.096	0.137
**AD-FOXN1**	18m HFD	d2	18.23	14.81	21.64	1.72	97.00		0.004	0.002
**AD-FOXN1**	18m HFD	d4	10.27	7.31	13.22	1.49	97.00			0.998
**AD-FOXN1**	18m HFD	d7	9.92	6.97	12.88	1.49	97.00			
**AD-GFP**	2m LFD	d0	9.84	6.88	12.80	1.49	97.00	0.924	0.977	0.850
**AD-GFP**	2m LFD	d2	11.16	8.20	14.12	1.49	97.00		0.730	0.998
**AD-GFP**	2m LFD	d4	8.98	6.02	11.94	1.49	97.00			0.617
**AD-GFP**	2m LFD	d7	11.55	8.59	14.50	1.49	97.00			
**AD-GFP**	2m HFD	d0	6.89	4.25	9.54	1.33	97.00	0.984	0.969	1.000
**AD-GFP**	2m HFD	d2	6.18	3.22	9.14	1.49	97.00		1.000	0.987
**AD-GFP**	2m HFD	d4	5.99	3.04	8.95	1.49	97.00			0.973
**AD-GFP**	2m HFD	d7	6.90	3.94	9.85	1.49	97.00			
**AD-GFP**	18m LFD	d0	11.22	8.26	14.18	1.49	97.00	<0.001	0.590	0.783
**AD-GFP**	18m LFD	d2	20.67	17.25	24.08	1.72	97.00		<0.001	<0.001
**AD-GFP**	18m LFD	d4	8.56	5.61	11.52	1.49	97.00			0.980
**AD-GFP**	18m LFD	d7	9.34	6.69	11.98	1.33	97.00			
**AD-GFP**	18m HFD	d0	5.33	2.38	8.29	1.49	97.00	<0.001	0.686	0.820
**AD-GFP**	18m HFD	d2	16.86	13.44	20.27	1.72	97.00		<0.001	<0.001
**AD-GFP**	18m HFD	d4	7.67	4.71	10.62	1.49	97.00			0.995
**AD-GFP**	18m HFD	d7	7.17	4.21	10.12	1.49	97.00			

Table S6. *Zfp521* mRNA expression in DFs with keratinocytes coculture—comparisons by day.

METHOD	GROUP	TIME	LSMEAN	LOWER.CL	UPPER.CL	SE	DF	D2	D4	D7
**AD-FOXN1**	2m LFD	d0	25.29	20.98	29.59	2.17	97.00	0.997	<0.001	<0.001
**AD-FOXN1**	2m LFD	d2	25.94	21.64	30.24	2.17	97.00		<0.001	<0.001
**AD-FOXN1**	2m LFD	d4	6.97	3.12	10.82	1.94	97.00			0.712
**AD-FOXN1**	2m LFD	d7	4.05	0.20	7.90	1.94	97.00			
**AD-FOXN1**	2m HFD	d0	21.24	17.39	25.08	1.94	97.00	0.005	0.026	0.002
**AD-FOXN1**	2m HFD	d2	11.27	6.97	15.57	2.17	97.00		0.950	0.958
**AD-FOXN1**	2m HFD	d4	12.91	8.61	17.21	2.17	97.00			0.750
**AD-FOXN1**	2m HFD	d7	9.60	4.63	14.57	2.50	97.00			
**AD-FOXN1**	18m LFD	d0	17.62	13.32	21.92	2.17	97.00	0.897	0.001	<0.001
**AD-FOXN1**	18m LFD	d2	15.48	11.18	19.78	2.17	97.00		0.011	<0.001
**AD-FOXN1**	18m LFD	d4	5.76	1.46	10.07	2.17	97.00			0.856
**AD-FOXN1**	18m LFD	d7	3.32	−0.98	7.62	2.17	97.00			
**AD-FOXN1**	18m HFD	d0	12.11	7.81	16.41	2.17	97.00	0.811	0.065	0.018
**AD-FOXN1**	18m HFD	d2	15.05	10.09	20.02	2.50	97.00		0.010	0.002
**AD-FOXN1**	18m HFD	d4	4.42	0.12	8.72	2.17	97.00			0.964
**AD-FOXN1**	18m HFD	d7	2.95	-1.35	7.26	2.17	97.00			
**AD-GFP**	2m LFD	d0	25.29	20.98	29.59	2.17	97.00	0.715	<0.001	<0.001
**AD-GFP**	2m LFD	d2	28.53	24.23	32.84	2.17	97.00		<0.001	<0.001
**AD-GFP**	2m LFD	d4	8.44	4.13	12.74	2.17	97.00			0.954
**AD-GFP**	2m LFD	d7	6.85	2.54	11.15	2.17	97.00			
**AD-GFP**	2m HFD	d0	21.24	17.39	25.08	1.94	97.00	0.013	0.093	<0.001
**AD-GFP**	2m HFD	d2	12.20	7.90	16.51	2.17	97.00		0.893	0.748
**AD-GFP**	2m HFD	d4	14.38	10.08	18.68	2.17	97.00			0.322
**AD-GFP**	2m HFD	d7	9.13	4.82	13.43	2.17	97.00			
**AD-GFP**	18m LFD	d0	17.62	13.32	21.92	2.17	97.00	0.557	0.002	<0.001
**AD-GFP**	18m LFD	d2	21.97	17.00	26.93	2.50	97.00		<0.001	<0.001
**AD-GFP**	18m LFD	d4	6.03	1.73	10.34	2.17	97.00			0.920
**AD-GFP**	18m LFD	d7	4.18	0.34	8.03	1.94	97.00			
**AD-GFP**	18m HFD	d0	12.11	7.81	16.41	2.17	97.00	0.373	0.130	0.041
**AD-GFP**	18m HFD	d2	17.47	12.50	22.44	2.50	97.00		0.002	<0.001
**AD-GFP**	18m HFD	d4	5.36	1.06	9.66	2.17	97.00			0.961
**AD-GFP**	18m HFD	d7	3.85	−0.45	8.16	2.17	97.00			

Table S7. List of antibodies used for flow cytometry assay.

Flow Cytometry Assay				
**Antibody**	**Probe Fluorochrome**	**Vendor**	**Cat. No**	**Clone**
CD3	PE	BD Biosciences	557749	SP34-2
CD31	PE	BD Biosciences	561073	MEC 13.3
CD45	PE	BD Biosciences	560695	104
CD24	APC	BD Biosciences	562349	M1/69
Sca1 (Ly-6A/E)	PE-Cy7	BD Biosciences	558162	D7
CD26	FITC	BD Biosciences	559652	H194-112
CD26	PE-Cy7	Biolegend	137809	H194-112
PDGFα	FITC	BD Biosciences	558774	APA5

Table S8. List of TagMan gene expression assays.

Gene	TaqMan® Pimer and Probe Sets ID
Pparγ	Mm00440940
Cebpα	Mm01265914_s1
Fabp4	Mm00445878_m1
leptin	Mm00434759_m1
Bmp2	Mm01340178_m1
Igf2	Mm00439564_m1
Zfp 521	Mm00521009_m1
Zfp 423	Mm00473699_m1
Foxn1	Mm01298129
Hprt1	Mm01545399_m1

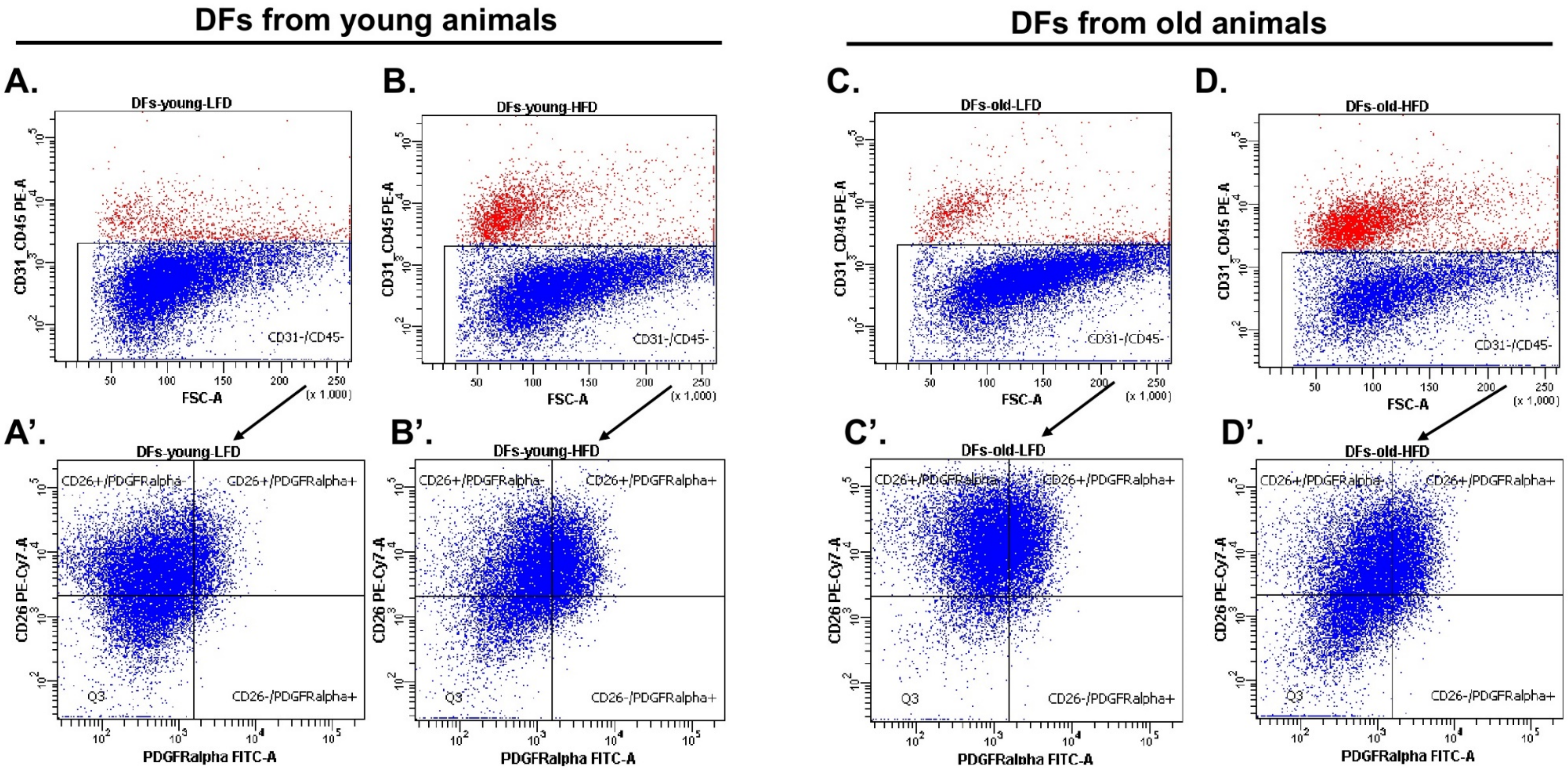

Figure S1

Flow cytometry analysis of DFs isolated from the skin of young, old, LFD or HFD C56BL/6(B6) mice. Representative dot-plots for Lin- (CD31-/CD45-) population in a group of DFs isolated from (A) young LFD, (B) young HFD, (C) old LFD, (D) old HFD mice. Further separation of CD26⁺ (CD31-/CD45-) and/ or PDGFRα⁺ (CD31-/CD45-) population in DFs isolated from (A') young LFD, (B') young HFD, (C') old LFD, (D') old HFD mice. The data are representative of n = 3–5 animals per age and diet. HFD, high-fat diet; LFD, low-fat diet.

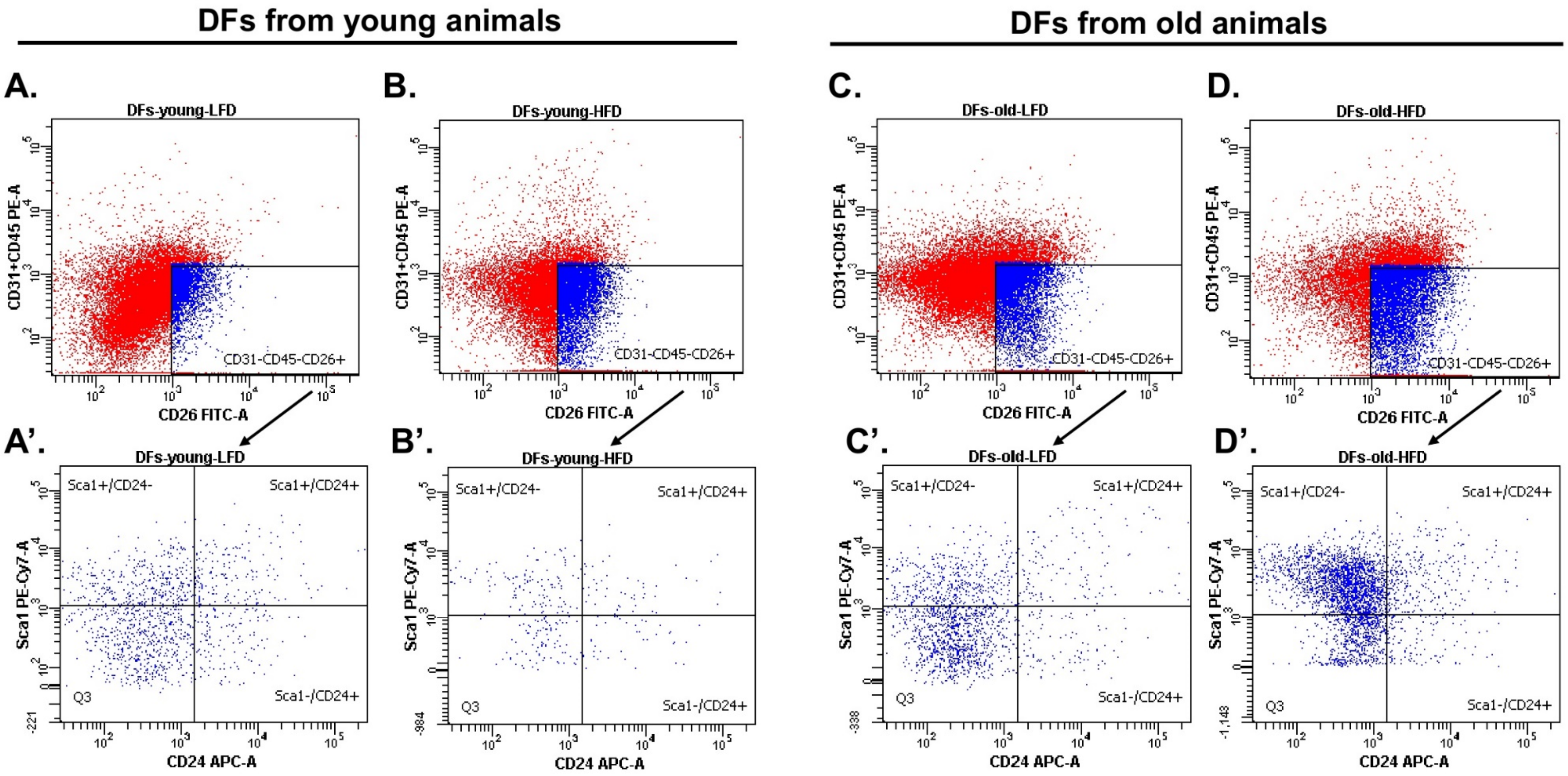

Figure S2

Representative dot-plots of $CD26^+$ cells within Lin- population in DFs isolated from (A) young LFD, (B) young HFD, (C) old LFD, (D) old HFD mice. $Sca1^+$, $CD24^+$ and $Sca1^+/CD24^+$ cells within $CD26^+$ population of DFs isolated from (A') young LFD, (B') young HFD, (C') old LFD, (D') old HFD mice. The data are representative of n = 3–5 animals per age and diet. HFD, high-fat diet; LFD, low-fat diet.

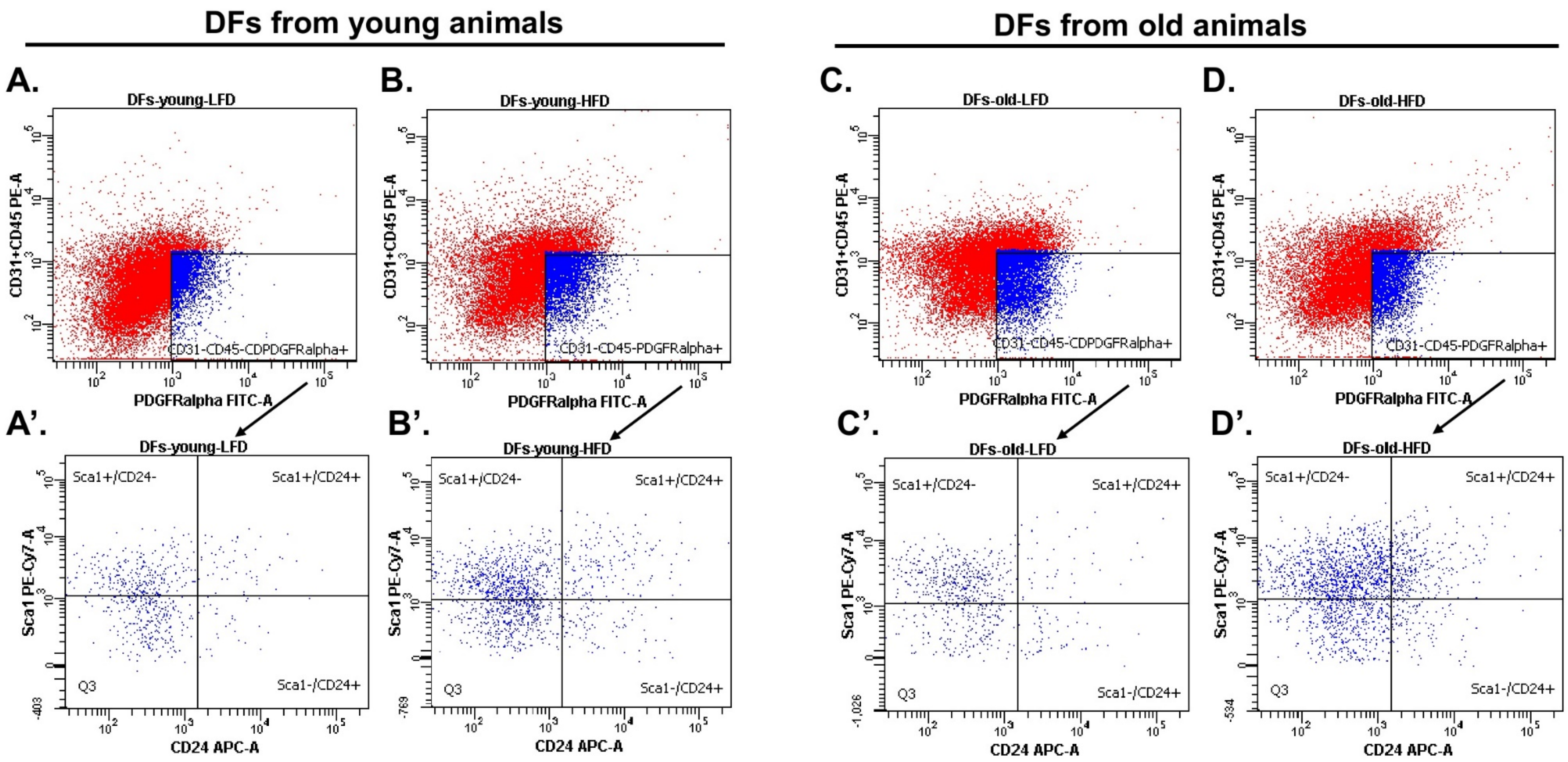

Figure S3

Representative dot-plots of PDGFRα⁺ cells within Lin- population in DFs isolated from (A) young LFD, (B) young HFD, (C) old LFD, (D) old HFD mice. $Sca1^+$, $CD24^+$ and $Sca1^+/CD24^+$ cells within $PDGFR\alpha^+$ population of DFs isolated from (A') young LFD, (B') young HFD, (C') old LFD, (D') old HFD mice. The data are representative of n = 3–5 animals per age and diet. HFD, high-fat diet; LFD, low-fat diet.
